# Androgen Deprivation Benefits in Low-Dose-Rate Brachytherapy With Hydrogel Spacer

**DOI:** 10.7759/cureus.68013

**Published:** 2024-08-28

**Authors:** Koyo Kikuchi, Shuhei Ishii, Yoshiro Ieko, Takafumi Segawa, Ryuji Nakamura, Hisanori Ariga

**Affiliations:** 1 Department of Radiation Oncology, Iwate Medical University, Morioka, JPN; 2 Department of Urology, Iwate Medical University, Morioka, JPN; 3 Department of Radiation Oncology, Iwate Prefectural Central Hospital, Morioka, JPN; 4 Department of Radiology, Morioka Red Cross Hospital, Morioka, JPN

**Keywords:** rectal bleeding, prostatic neoplasms, hydrogel spacer placement, brachytherapy, androgen antagonists

## Abstract

Introduction: We aim to investigate the impact of rectal dose reduction of both androgen deprivation therapy (ADT) and concurrent hydrogel spacer placement (HSP) in patients treated with low-dose-rate (LDR) brachytherapy for prostate cancer and to determine whether there are variations in the degree of efficacy of dose reduction across different segments of the rectum.

Methods: This study involved 130 consecutive patients treated with I-125 LDR brachytherapy, with (ADT: n = 66) or without (nADT: n = 64) prior ADT, from June 2017 to April 2021. Among these, 13 ADT and 17 nADT patients underwent HSP following induction in May 2020, whereas the remaining patients (nHSP) included 53 ADT and 47 nADT individuals. In the post plan, a rectal dose assessment was made using the rectal volume (RV), divided by horizontal sections into three equal-length subparts (sRVs), such as high-, mid-, and low-RV. The mean sRV_100_ values were compared between the nADT and ADT patient groups, both with and without HSP. Similarly, mean sRV_100_ was compared between the nHSP and HSP patient groups, both with and without ADT.

Results: In nADT patients, HSP significantly reduced the mean RV_100_ of the high-RV (0.002 cc versus 0.086 cc, p < 0.05) and mid-RV (0.127 cc versus 0.377 cc, p < 0.05), but not of the low-RV (0.060 cc versus 0.150 cc, p = 0.06). In contrast, in ADT patients, HSP significantly reduced the RV_100_ at all three sites (0.002 cc versus 0.031 cc, p < 0.05; 0.034 cc versus 0.269 cc, p < 0.05; and 0.015 cc versus 0.151 cc, p < 0.05, respectively). No significant difference was observed when comparing mean sRV_100_ with or without ADT in both HSP and nHSP patients.

Conclusion: The combination of ADT and HSP for LDR prostate brachytherapy showed the potential to significantly reduce RV_100_, especially in the lower rectum.

## Introduction

Low-dose-rate (LDR) brachytherapy is a standard treatment for localized prostate cancer, which is frequently combined with androgen deprivation therapy (ADT) to achieve hypertrophic prostate volume reduction for brachytherapy availability [1] or to decrease biochemical failure and disease-specific mortality in the long-term follow-up for high-risk prostate cancer [2]. When external beam radiotherapy (EBRT) is combined with brachytherapy and ADT, it is a guideline-concordant optimal multimodality therapy for high-risk prostate cancer [3].

The prevailing late adverse effects of prostate radiotherapy include rectal comorbidities. The most common manifestation of chronic radiation proctitis is anterior rectal wall bleeding, which often occurs within the first two years of radiotherapy [4]. Even after a modern LDR brachytherapy, the incidence of late rectal adverse events equal to or more than grade 2 was 8% [5]. Supplemental EBRT may lower the threshold for rectal toxicity after LDR brachytherapy [6]. The three-year cumulative incidence rates of equal to or more than grade 2 late rectal toxicity in LDR brachytherapy and LDR brachytherapy combined with EBRT were 0.90% and 5.01%, respectively [7]. Comorbidities considerably compromise the quality of life of patients due to refractory episodes or non-negligible burden by necessitating close examination, such as colonoscopy, for the denial of coexistent colon cancer. Owing to many efforts to minimize toxicity, a close relationship has been revealed between rectal bleeding rates and dose-volume histogram (DVH) variables using the radiotherapy plan. There are various recommendations regarding DVH parameters related to rectal bleeding after LDR brachytherapy. The American Brachytherapy Society consensus statement recommends that the rectal volume receiving 100% of the prescribed dose (RV_100_) should be less than 1 cc for post-implant dosimetry [8]. The Groupe Européen de Curiethérapie-European Society for Radiotherapy & Oncology (GEC-ESTRO) Advisory Committee for Radiation Oncology Practice (ACROP) prostate brachytherapy guidelines suggest that the rectum D_2 cc_ should be less than 145 Gy and D_0.1 cc_ should be less than 200 Gy [9]. Among these parameters, RV_100_ has been reported as an indicator for predicting rectal bleeding in LDR brachytherapy [10-14].

Temporary hydrogel spacer placement (HSP) between the prostate and rectum has been increasingly used to reduce the risk of rectal bleeding [15]. In HSP, it is necessary for the connective tissues lying between the prostate and rectum to be exfoliated by saline to secure the space embedded by the spacer; however, this procedure is sometimes more difficult to achieve on the caudal side than on the cephalic side. We noticed that this was not the case when patients had already been treated with ADT.

It is well known that ADT reduces prostate volume [16,17]; however, its effects on other DVH variables in LDR brachytherapy have rarely been studied. The objective of this study is twofold: firstly, to investigate the impact of RV_100_ reduction in brachytherapy patients with ADT and HSP; and secondly, to ascertain whether there are variations in the degree of efficacy of dose reduction across the various segments of the rectum.

## Materials and methods

Patients

This single-center, retrospective study was approved by the Institutional Review Board of Iwate Medical University (approval number: MH2021-121) and has, therefore, been performed in accordance with the ethical standards laid down in the Declaration of Helsinki. Informed consent was obtained through the opt-out form on the website. The study analyzed 130 consecutive patients with prostate LDR brachytherapy between June 2017 and April 2021. The inclusion criteria were adult patients with biopsy-proven prostate cancer and an Eastern Cooperative Oncology Group performance status of 0 or 1. The exclusion criteria were transurethral resection of the prostate and inflammatory bowel disease requiring treatment with steroids, lupus, and scleroderma. Sixty-six patients received ADT pre-treatment (ADT patients), while 64 did not (nADT patients). Table 1 shows the backgrounds of the patients. At our institution, HSP was initiated in May 2020. Out of the 130 patients, 70 patients who received brachytherapy before May 2020 did not undergo HSP (nHSP patients), while all 30 patients who received brachytherapy after May 2020 underwent HSP (HSP patients). The study divided patients into four subgroups based on the presence or absence of ADT and HSP (Table 2).

**Table 1:** Patients' background Abbreviations: (n)ADT: (non-)androgen deprivation therapy, PSA: prostate-specific antigen, EBRT: external beam radiotherapy, PV_100_: prostate volume of those receiving 100% of the prescribed dose, RV_100_: rectum volume of those receiving 100% of the prescribed dose, RD_2 cc_: minimum dose received by 2 cc of the rectum

	nADT patients (n = 64)	ADT patients (n = 66)	All (N = 130)
Mean age	66	68	67
T stage			
T1	27	20	47
T2	37	39	76
T3	0	7	7
Initial PSA (ng/ml) (mean)	7.69	9.32	8.52
Gleason grade group			
1-2	55	28	83
3	9	8	17
4-5	0	30	30
Prescription dose			
110 Gy with EBRT	10	40	50
160 Gy	54	26	80
Number of I-125 seeds (mean)	74.4	55.0	64.6
PV_100_ (mean)	96.6%	94.3%	95.4%
RV_100_ (mean)	0.50 cc	0.37 cc	0.44 cc
RD_2 cc_ (mean)	102.2 Gy	79.5 Gy	90.6 Gy
RD_0.1 cc_ (mean)	172.7 Gy	144.6 Gy	158.5 Gy

**Table 2:** Number of patients in four subgroups Abbreviations: ADT: androgen deprivation therapy, HSP: hydrogel spacer placement

		ADT prior to brachytherapy		Total
		No	Yes	
HSP	No	47	53	100
	Yes	17	13	30
Total		64	66	130

Radiotherapy

All the patients were irradiated with either brachytherapy alone or brachytherapy plus EBRT. The prescribed dose of brachytherapy or EBRT and the necessity for ADT were determined in accordance with the National Comprehensive Cancer Network guideline as follows: for patients with a low to favorable intermediate risk, brachytherapy of 160 Gy alone; for an unfavorable intermediate risk, brachytherapy of 110 Gy and supplemental EBRT of 40-50 Gy in 20-25 fractions to achieve a combined dose of 200 Gy as the biologically effective dose in α/β = 2 (BED2); and for high risk, brachytherapy (110 Gy), EBRT (40-50 Gy in 20-25 fractions to achieve a combined dose of 220 Gy (BED2)), and ADT starting from six months prior to brachytherapy to 24 months after brachytherapy. Patients with prostate volume of 40 cc or greater were also treated with ADT to reduce the volume before brachytherapy for a few months.

Brachytherapy was performed using I-125 linked seeds (0.28-0.335 mCi; SourceTec I-125 NIST99; Bard, Murray Hill, NJ) inserted with a Mick Applicator (Mick Radio-Nuclear Instruments, Mount Vernon, NY). Modified peripheral loading was optimized via transrectal ultrasound-guided real-time interactive implantation, with the dose constraints such as prostate volume (PV) receiving 100% of the prescribed dose (PV_100_) > 99%, PV receiving 150% of the prescribed dose (PV_150_) < 60%, and RV_100_ <1 cc, urethral volume (UV) receiving 150% of the prescribed dose (UV_150_) < 0.1 cc. After seed implantation, a transrectal ultrasound-guided perineal puncture was performed, and the connective tissue between the prostate and rectum was dissected with approximately 5-10 cc of saline, followed by placement of a commercial hydrogel spacer (SpaceOAR, Boston Scientific, Marlborough, MA), as described previously [18].

**Figure 1:**
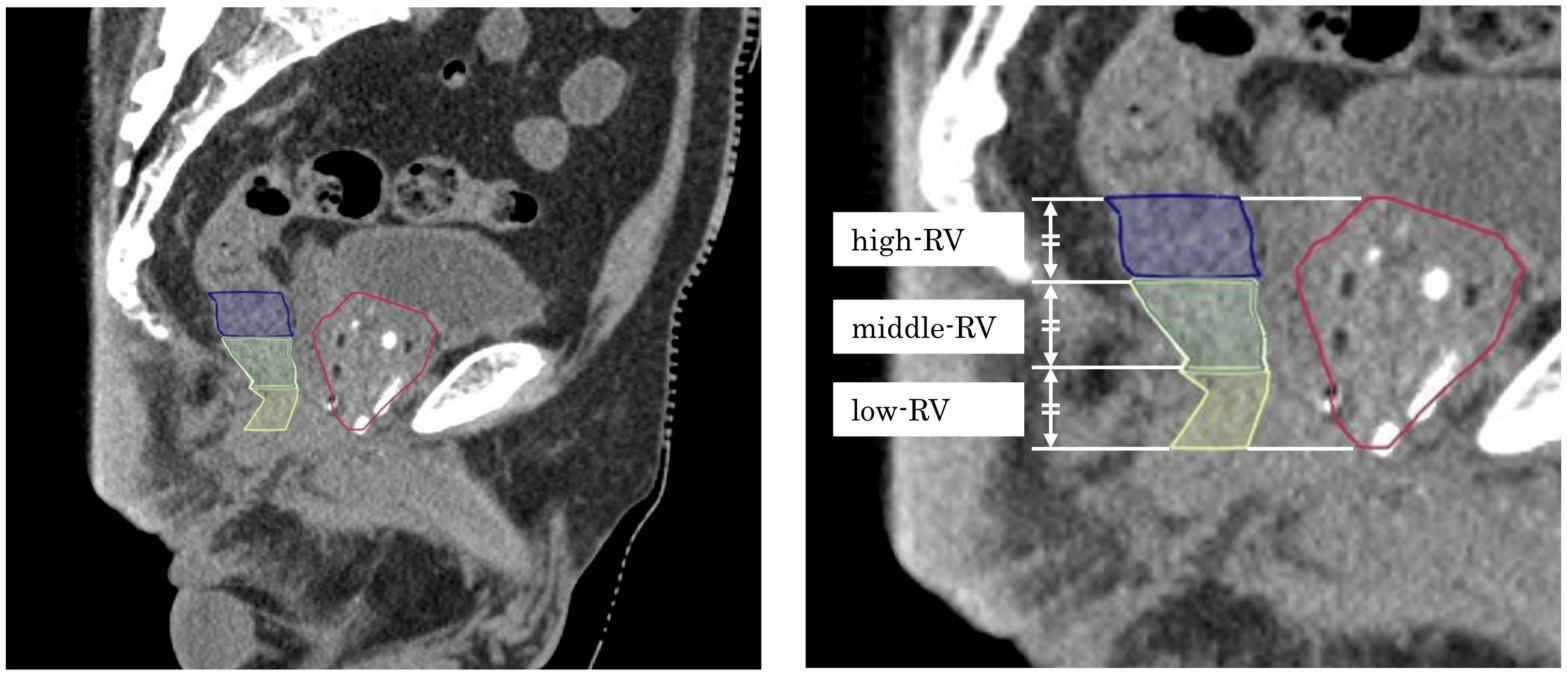
The rectum was defined as a solid structure representing the outer contour of the section containing the prostate (red). RV was divided into three equal-length subparts: high-RV (blue), middle-RV (green), and low-RV (red). Abbreviation: RV: rectal volume

Post plan was performed using computed tomography 30 days after brachytherapy using a treatment planning system (VariSeed version 8, Varian Medical Systems, Palo Alto, CA). The DVH indices, including the PV, RV, and RV_100_, were extracted from the DVH indices routinely calculated for estimating radiotherapy. The rectum is defined as a solid structure comprising the outer contour of the slice in which the prostate is located. In this study, the RV was divided into three equal-length subparts (sRV), such as high-, middle-, and low-RV (Figure 1). RV and RV_100_ were calculated individually in each of the sRVs.

Study design and data analysis

The mean PV was compared between the ADT and nADT patients to estimate the impact of ADT on PV.

Comparison of sRV_100_ between subgroups revealed the influence of confounding cohort modalities. The mean sRV_100_ values were compared between the nADT and ADT patient groups, both with and without HSP. Similarly, in the reverse stratification, the mean sRV_100_ was compared between the nHSP and HSP patient groups, both with and without ADT.

The mean values were compared using the Student's t-test for statistical significance. A p-value of <0.05 was considered to indicate statistical significance. IBM SPSS statistics software version 26 (IBM Corp, Armonk, NY) was used for all analyses.

## Results

All patients underwent brachytherapy with intraoperative DVH indices that satisfied the required thresholds. ADT, composed of bicalutamide and a luteinizing hormone-releasing hormone agonist, was administered for a mean of six months (range: 2-11) prior to brachytherapy. A total of 63 patients received ADT for 4-11 months. Both brachytherapy and brachytherapy followed by HSP were successfully performed, and no acute adverse effects equal to or greater than grade 2 occurred. The interspaces created by the spacer varied in distance along the longitudinal axis of the rectum. No significant dislocation of the spacer, such as infiltration into the rectum or discontinuity of the muscularis propria, was observed [19].

ADT patients had a smaller mean PV (22.0 cc; range: 10.7-38.1 cc) compared to nADT patients (26.9 cc; range: 14.9-42.2 cc) (p < 0.05) (Table 3). As the length of RV was determined by that of PV, the mean RV of ADT patients (20.0 cc; range: 8.6-52.4 cc) was decreased compared to that of nADT patients (27.1 cc; range: 10.7-72.1 cc) (p < 0.05), in proportion to the rate of collateral PV reduction due to ADT (Table 4). When the mean PV and RV were compared separately between HSP and nHSP patients, no significant differences were observed (p = 0.57, p = 0.12).

**Table 3:** ADT and HSP effects on mean prostate volume (cc) Abbreviations: (n)ADT: (non-)androgen deprivation therapy, (n)HSP: (non-)hydrogel spacer placement

	Mean prostate volume		Total	p-value
	nADT patients	ADT patients		
nHSP patients	26.4	23.1	24.6	p = 0.57
HSP patients	28.3	17.8	23.7	
Total	26.9	22.0		
	p < 0.05			

**Table 4:** ADT and HSP effects on mean rectum volume (cc) Abbreviations: (n)ADT: (non-)androgen deprivation therapy, (n)HSP: (non-)hydrogel spacer placement

	Mean rectum volume		Total	p-value
	nADT patients	ADT patients		
nHSP patients	27.7	21.2	24.3	p = 0.12
HSP patients	25.1	15.1	20.8	
Total	27.1	20.0		
	p < 0.05			

In the group of nADT patients, the mean sRV_100_ of those with HSP (n = 17) was decreased compared to that of those without HSP (n = 47) in the high-RV (0.002 cc versus 0.086 cc) (p < 0.05) and in the middle-RV (0.127 cc versus 0.377 cc) (p < 0.05), but not in the low-RV (0.060 cc versus 0.150 cc) (p = 0.06) (Table 5). In contrast, in the group of ADT patients with HSP (n = 13), the mean value of sRV_100_ was decreased in all sRVs compared to that of those without HSP (n = 53) (high-RV: 0.002 cc versus 0.031 cc (p < 0.05), middle-RV: 0.034 cc versus 0.269 cc (p < 0.05), and low-RV: 0.015 cc versus 0.151 cc (p < 0.05)) (Table 6).

**Table 5:** Dose-volume effects on the rectum in nADT patients *Statistically significant Abbreviations: (n)ADT: (non-)androgen deprivation therapy, (n)HSP: (non-)hydrogel spacer placement, (s)RV: (subparts) rectum volume, (s)RV_100_: (subparts) rectum volume of those receiving 100% of the prescribed dose

	nADT patients (n = 64)	mean sRV (cc)			mean sRV_100_ (cc)		
		high-RV	middle-RV	low-RV	high-RV	middle-RV	low-RV
HSP	No (n = 47)	12.29	9.21	6.24	0.086	0.377	0.150
	Yes (n = 17)	12.08	7.98	5.07	0.002	0.127	0.060
% of decline		1.7%	13.4%	18.8%	97.7%	66.3%	60%
p		0.92	0.32	0.07	<0.05*	<0.05*	0.06

**Table 6:** Dose-volume effects on the rectum in ADT patients *Statistically significant Abbreviations: (n)ADT: (non-)androgen deprivation therapy, (n)HSP: (non-)hydrogel spacer placement, (s)RV: (subparts) rectum volume, (s)RV_100_: (subparts) rectum volume of those receiving 100% of the prescribed dose

	ADT patients (n = 66)	mean sRV (cc)			mean sRV_100_ (cc)		
		high-RV	middle-RV	low-RV	high-RV	middle-RV	low-RV
HSP	No (n = 53)	8.96	6.76	5.48	0.031	0.269	0.151
	Yes (n = 13)	6.42	4.91	3.81	0.002	0.034	0.015
% of decline		28.3%	27.4%	30.5%	93.5%	87.4%	90.1%
p		<0.05*	<0.05*	<0.05*	<0.05*	<0.05*	<0.05*

No significant difference in sRV_100_ was observed in the nHSP patients group (Table 7) or in the HSP patients group, with or without ADT (Table 8).

**Table 7:** Dose-volume effects on the rectum in nHSP patients *Statistically significant Abbreviations: (n)ADT: (non-)androgen deprivation therapy, (n)HSP: (non-)hydrogel spacer placement, (s)RV: (subparts) rectum volume, (s)RV_100_: (subparts) rectum volume of those receiving 100% of the prescribed dose

	nHSP patients (n = 100)	sRV (cc)			sRV_100_ (cc)		
		high-RV	middle-RV	low-RV	high-RV	middle-RV	low-RV
ADT	No (n = 47)	12.29	9.21	6.24	0.086	0.377	0.150
	Yes (n = 53)	8.96	6.76	5.48	0.031	0.269	0.151
% of decline		27.1%	26.6%	12.2%	64%	28.6%	-0.7%
p		<0.05*	<0.05*	0.07	0.07	0.09	0.98

**Table 8:** Dose-volume effects on the rectum in HSP patients *Statistically significant Abbreviations: (n)ADT: (non-)androgen deprivation therapy, (n)HSP: (non-)hydrogel spacer placement, (s)RV: (subparts) rectum volume, (s)RV_100_: (subparts) rectum volume of those receiving 100% of the prescribed dose

	HSP patients (n = 30)	sRV (cc)			sRV_100_ (cc)		
		high-RV	middle-RV	low-RV	high-RV	middle-RV	low-RV
ADT	No (n = 17)	12.08	7.98	5.07	0.002	0.127	0.060
	Yes (n = 13)	6.42	4.91	3.81	0.002	0.034	0.015
% of decline		46.9%	38.5%	24.9%	0%	73.2%	75%
p		<0.05*	<0.05*	<0.05*	0.85	0.16	0.20

## Discussion

To clarify the impact of ADT or HSP on the RV_100_, a complicated stratification of patients is required since both may affect the RV_100_. The RV was divided into three subparts, and the RV_100_ of each sRV part was individually compared. A meticulous analysis revealed that antecedent ADT induced a caudal increase in the protective effects of spacer placement. The observed effect is in line with our previous findings. Adequate spacer placement at the caudal part of the interspace after ADT administration is feasible. On the other hand, ADT did not experience a significant decrease in sRV_100_ in both the nHSP and HSP patient groups. This suggests that ADT alone may not effectively reduce RV_100_. To the best of our knowledge, no research has reported a favorable effect of ADT on rectal dosimetry in widely used radiotherapy for prostate cancer.

As spacer placement is performed after brachytherapy completion, it is not an interactive procedure, in contrast to intraoperative brachytherapy planning. The predictive variables for RV were not revealed until the post plan one month later. In this regard, prejudice regarding the fragile spacing effect around the caudal part of the rectum is noteworthy for brachytherapy as monotherapy. However, this is not the case in the brachytherapy plus EBRT strategy because EBRT performed afterward is able to regulate the rectal exposure dose, to some extent, according to that of the previous brachytherapy [20].

The hydrogel spacer may not be fully implanted caudally due to anatomical constraints around the prostatic apex. The fatty tissue between the Denonvilliers fascia and the rectal wall is sufficient on the cephalic side but tight on the caudal side due to the space between the prostate apex and the levator ani muscle. ADT reduced the volume of the prostate and likely loosened the space between the apex of the prostate and the levator ani muscle, even caudally, facilitating saline dissection. By successfully performing HSP in the caudal position, HSP patients may experience a decrease in RV_100_ in the low-RV.

The various adverse effects induced by long-term ADT are well-known clinical entities that are compensated for by their oncological contributions. They include systemic effects outside of the prostate, such as fatty or connective tissue that is constitutionally distributed. Markiewicz et al. [21] found that androgens play a positive role in the regulation of collagen biosynthesis and that castration markedly decreases skin thickness and collagen content in mice. Santosa et al. [22] reported that even acute testosterone withdrawal (49 days) promoted subcutaneous abdominal and femoral adipose tissue fatty acid storage in human volunteers. These constitutional changes may have also occurred in the interspace tissue, which could reduce its resistance to exfoliation by saline.

This was a retrospective study provoked by the finding of longitudinally asymmetric expansion of the spacer along the posterior wall of the prostate in patients treated with multiple confounding modalities. One of the limitations of this study is the relatively small and imbalanced number of patients distributed in each subgroup for the comparison of the rectal dose. The comparison of patient variables stratified by modality had less certainty compared to that of the multivariate analysis.

## Conclusions

HSP reduces the dose to the rectum in LDR brachytherapy, even in the absence of prior ADT. However, the efficacy of this approach is enhanced when combined with ADT, particularly in reducing the dose to the lower rectum. Further studies are needed to elucidate the impact of this strategy on rectal toxicity.
